# The Causes of Premature Baldness

**Published:** 1878-10

**Authors:** George H. Rohe

**Affiliations:** Atlanta


### THE CAUSES OF PREMATURE BALDNESS.

By GEORGE H. ROHE, M.D., Atlanta.

The great prevalence of early baldness among Americans is a fact daily forced upon the attention of every one whether physician or layman. While itself never fraught with serious consequences, it is yet very annoying, and when affecting the gentler sex, causes frequently such disfigurement as to compel the wearing of artificially arranged tresses, braids, chignons, or by whatever names they may be known.

It is the object of this paper to call attention to some of the causes of this premature falling of the hair, and to suggest remedies for its prevention.

The hair-sheath is nothing but an infolding of the epidermic layer of the skin, having its lower end somewhat expanded, and forming a bottle-shaped sac, from the bottom of which the hair takes its origin. Near the point where the hair-sheath opens upon the skin, there is usually found a small opening in its walls, which is the mouth of a sebaceous gland. The mouth of the hair-sheath then, is the point of exit, both of the hair and of the secretion of one or more of the sebaceous glands, the function of whose secretion it is to keep the skin and hairs soft and pliant.

Now, in certain morbid conditions of the body, either general or local—more usually perhaps the former—the secretion of the sebaceous glands is increased in quantity and altered in quality, becoming more fluid or more solid than normal. Under these circumstances we have the affection technically termed seborrhœa, one form of which—and the only one that concerns us here—is commonly known as "dandruff."

In the older works on skin diseases, dandruff is usually described as a scaling of the superficial layers of the epidermis. More recent investigations, however, have shown that, in the vast majority of cases, at least, it consists in a dry seborrhœa, *i. e.* in increase of sebaceous secretion of an altered character.

This altered sebaceous matter, not finding such ready exit on account of its abnormal dryness, chokes up the hair-sheath, and finally—generally after months or years—leads to falling of the hair and atrophy of the hair bulb.

Any one may easily convince himself of the fact that the scaly matter in dandruff is the altered secretion of the sebaceous glands and not merely a desquamation of the epidermis, by a very simple experiment. If after "shampooing" the head to remove all the scales, a dilute solution of glycerine in water be used on the head once or twice a day, the scales will not re-form as rapidly as before, but in a few days small, hardened plugs of a yellowish, waxy matter, will be found about the base of the hairs, which may be easily removed with the finger nail. The glycerine prevents the rapid desiccation of the sebum, and so gives rise to the formation of plugs instead of scales. These plugs are similar to those sometimes found in comedo, with the exception of being firmer in consistence.

A distinguished French microscopist, M. Malassez, claims that dandruff and alopecia are due to a vegetable parasite, the spores of which he has found in great numbers among the scales. As this discovery has not been corroborated, we may regard it as open to question. It is worthy of notice, however, that M. Malassez recommends similar treatment to that proposed below.

The alopecia consequent on the specific fevers, and other grave constitutional diseases is usually preceded and accompanied by a more or less general seborrhœa. This is especially marked in typhoid fever, syphilis and chronic pulmonary or digestive diseases. In general debility, whether from overwork or other causes, there is very often a marked degree of general seborrhœa present, and it is likewise in such cases in which early loss of hair usually occurs.

Those cases in which patches of the scalp become suddenly entirely devoid of hair, known as alopecia areata, area celsi, etc., are not due to seborrhœa. It is also not probable, as claimed by some, that this variety is parasitic. All our present knowledge on the subject points to nutritive derangement, probably due to defective nervous supply of the hair, as the cause of this form of baldness.

Seborrhœa, then, being in the majority of cases the direct cause of loss of hair, it becomes important to know what remedies are available for the prevention or cure of this condition. When we consider that the causes of this trouble are very frequently constitutional, it will be at once seen that local remedies alone are not to be depended upon. Thus no one could expect to permanently cure the seborrhœa of constitutional syphilis without taking account of the general disease in the treatment. But, on the other hand, systemic remedies, if alone relied on, would nearly always disappoint any expectations of success. A judicious combination of general and local measures is, therefore, necessary for the attainment of the desired result, viz., the cure of the patient.

In all cases of premature alopecia, we should endeavor to determine whether there is any depression of vital power depending either on some grave constitutional disease, as syphilis or tuberculosis, or more commonly on innutrition from chronic dyspepsia. Tonics, good food, fresh air, exercise and regular habits suggest themselves to every one. Of medicinal agents, iron and arsenic, which may be given in combination, and the mineral acids will prove the most useful. The latter are especially indicated when dyspepsia is present. In some cases cod liver oil and iodine are valuable remedies. It will be well to bear in mind, however, that none of these remedies are specifics in the affection under consideration, but only of value on account of their generally recognized therapeutic properties.

Most persons affected with dandruff are in the habit of removing the scales with a fine-tooth comb or a stiff hair brush. Both of these methods are objectionable, as they only increase the trouble by the irritation of the surface of the scalp and the glands which results from their use. A stiff hair-brush or sharp fine-tooth comb should, therefore, first of all, *not* be used.

Having been himself a sufferer from seborrhœa and consequent alopecia for six or seven years, the writer has, as may be supposed, tried a great many remedies with a view to its alleviation and cure. Arsenic internally, stimulating washes or oily applications, containing in the one case, corrosive sublimate, in the other quinine, or tannin; in still another some of the stimulating oils were used with no appreciable effect either on the formation of scales or the depilation. Finally, about two years ago, an item went the rounds of the medical journals to the effect that a French physician, whose name has escaped me, had found that the local use of a five per cent. solution of chloral hydrate was a sovereign remedy for the trouble under consideration. Rejoiced that at last I could appropriately shout "Eureka!" I began to use the chloral wash assiduously for about three months, following the directions given as accurately as possible. At the end of the three months, the production of scales was more rapid and the fall of hair greater than ever. Disgusted with the failure of all the therapeutic measures which had been so highly lauded, I almost decided to let the affection take its own course, and run the risk of a shiny bald pate at thirty. About that time the second volume of Hebra's classical treatise on diseases of the skin* came to hand, and one of

*Hebra & Kaposi; Hautkrankheiten, 2 Band. Erlangen, 1876.

the first things I read was Kaposi's thorough article on alopecia. Impressed with the reasonableness of the views put forth by Kaposi, I determined to give his plan of treatment a trial, with the result of checking the fall of hair and diminishing the production of scales in a reasonably short space of time. I have since then recommended the plan in a considerable number of instances, and when it has been faithfully carried out, with uniform success.

The success of the method depends upon the use of an agent which, while mildly stimulant, removes the scales and thoroughly cleanses the scalp. This agent is the German or French soft soap (green soap, schmierseife, savon vert.) in alcoholic solution. This soap is now imported in large quantities and prescribed daily by the dermatologists of Boston, New York, Baltimore, Philadelphia and other cities. The soap containing an excess of alkali saponifies the fatty matter of the sebaceous secretion, and it is thus easily removed. The alcohol greatly assists this action, and seems also to have an alterative action—if such an indefinite term is excusable—on the glands. The two may be combined, as follows:

℞—Saponis viridis, (Germ.),
Alcoholis, aa ℥ij.
M.—Solve, filtra, et adde
Ol. Lavandulæ gtt. xx—xxx.

The oil of lavender is added to cover the disagreeable fishy odor of the soap. The above makes a very handsome, orange or wine-colored preparation, with a pleasant odor, to which the most fastidious will hardly object.

This is used as a shampoo every morning or evening, pouring one or two tablespoonfuls on the head. Upon the addition of water, and smart friction with the fingers, a copious lather is soon produced. After keeping up the shampooing process for four or five minutes, all the soap must be washed out of the hair by the free use of warm or cold water, and the hair thoroughly dried by means of gentle friction with a soft towel. The immediate effect experienced is a disagreeable feeling of tension of the scalp, as if it were stretched too tightly over the skull. To obviate this effect, and to keep the scalp from getting too dry, and thus, perhaps, set up a true pityriasis, it is necessary to follow up the shampooing with some fatty application; which may contain some mild stimulant, thus: Castor oil, 1 part, to alcohol 3 or 4 parts, with a little oil of rosemary or cinnamon, or the elegant pomades and oils of Bazin and other manufacturers may be used. But the best, as well as the neatest preparation that I have employed for this purpose, is the hydro-carbon known in commerce as cosmoline. This is a product obtained from petroleum. It is entirely bland and unirritating; never turns rancid, and is comparatively cheap. It may be obtained in the fluid form or as a soft solid.

This procedure, shampooing, drying the hair and applying the greasy preparation must be repeated daily for three or four weeks. In the course of that time it will be discovered that the production of scales and the falling of the hair has been very markedly decreased. It will then suffice to repeat it twice or three times a week for a month or two longer, after which a good shampoo once a week will usually succeed in maintaining a permanent cure.

Most patients will be alarmed after using this method at first, because the hair comes out in greater quantity than before. This is due to the fact that a large number of hairs are dead and only retained in their follicles by the plugging of the sheath with the accumulated sebaceous matter. The patient should, therefore, always be prepared for this result, and the cause of the increased falling of the hair explained to him.

It is not necessary—though more convenient—to cut the hair short during the treatment.

When the alopecia has lasted so long that the hair-bulbs have become atrophied, nothing will restore the hair on those spots. Our endeavors must be directed to saving what remains. A prognosis favorable to the restoration of the hair must, therefore, be given with caution.

